# Performance enhancement in CZTSSe solar cells via BaSi₂ back surface field integration

**DOI:** 10.1038/s41598-025-31243-3

**Published:** 2025-12-05

**Authors:** T. R. S. Chandran, Deepak Kumar Panda, Pratikhya Raut, Amit Kumar Goyal

**Affiliations:** 1 Amrita School of Engineering Amaravati, Amrita Vishwa Vidyapeetham, Coimbatore, 522503 Andhra Pradesh India; 2 Department of ECE, School of Engineering, Siddhartha Academy of Higher Education, Vijayawada, 520007 Andhra Pradesh India; 3 Department of Electronics and Communication Engineering, Manipal Institute of Technology, Manipal Academy of Higher Education, Manipal, 576104 India

**Keywords:** Solar cell, Efficiency, CZTSSe, BSF, BaSi_2_, Clean energy, Sustainable energy, Electrical and electronic engineering, Renewable energy

## Abstract

The efficiency of solar cells is strongly influenced by factors such as durability, cost-effectiveness, environmental compatibility, and overall performance. Recent advancements in kesterite-based CZTSSe solar cells have revealed a persistent challenge of low open-circuit voltage (V_OC_), which significantly limits device efficiency. This work focuses on optimizing absorber and interface properties to enhance the simulated performance of CZTSSe solar cells. The thermal stability of the proposed structure is also evaluated by examining the effect of operating temperature on key photovoltaic parameters. To address performance limitations, a BaSi₂-based back surface field (BSF) layer is incorporated, and numerical simulations are carried out using the SCAPS-1D software. The introduction of the BaSi₂-based BSF layer effectively reduces V_OC_-related losses and enhances the overall device efficiency. The model’s validity is supported through comparison with previously published experimental and simulation data. Incorporating BaSi₂ as the BSF layer increases the simulated efficiency from 12.54% to 16.37%. In parallel, a systematic study of the CZTSSe absorber layer was conducted to determine the optimal thickness and doping concentration for further improving solar cell performance. The values can be varied systematically, such as the absorber’s layer thickness from 0.5 to 3 μm, and the doping concentration is modified from 10^12^ to 10^18^ cm^− 3^. An efficiency of 19.61% can be achieved for the recently improved configuration using a CZTSSe thickness of just only 0.5 μm under idealized conditions but not experimentally realistic. This reduction of the thickness of the CZTSSe solar cells is an important factor in the decline of performance, but it can improve the lifetime of minority carriers.

## Introduction

 The usage of renewable energy resources has increased in today’s daily life due to the consequences of climate change and also the increase in power consumption. The primary reason for using these sources is their sustainability, as they can be recharged. In the past years, it has been observed that Photovoltaic (PV) cell (also known as Solar cell) power generation plays an important role among the renewable energy sources. The operation behind a PV cell is converting solar energy into electrical energy by using semiconductor materials^1^. The research community^2^ has proposed and explored different materials for use in PV cells, but these cells will have a main advantage of giving a strong output at a cost-effective price.

One of the major types of solar cells is the Thin-Film Solar Cell (TFSC), which offers the benefits of conserving natural resources, the capability to absorb specific wavelengths of light, and high efficiency. Among TFSCs, Cadmium Telluride (CdTe) and Copper Indium Gallium Selenide (CIGS) are two major types that have achieved efficiencies of 24.2%^3^ and 23.6%^4^, respectively. Since these types of solar cells have some disadvantages, such as containing toxic elements, high expenses, and vital components that affect the progress of solar systems. Hence, a study on improvement in thin film solar cells^5^, which are fair, prudent, feasible, and financially viable devices.

Kesterite semiconductors, which are influenced by chalcopyrite CIGS by switching Zn and Sn for In and Ga, accordingly, have received more attention^6,7^. The kesterite CZTSSe has become as a feasible option to all present marketed thin-film technologies^8,9^. CZTSSe was an important alternative as an absorber component in TFSCs, as it has a good absorption coefficient (> 10^4^ cm^− 1^), p-type conductivity, and a direct and narrow band gap (1.0–1.5.0.5 eV). The considered material CZTSSe is a Se-rich material, and hence the bandgap of CZTSSe is closer to 1.0eV^45^. The materials present in CZTSSe are widely available, abundant on Earth, and eco-friendly^10^.

The CZTSSe is playing an important role in the growth of thin film-based photovoltaic technology due to its power conversion efficiency (PCE), which has increased from approximately 5% in 2004 to 13.6%^11^ and 14.9% in 2023^12^. The Performance gap between CZTSSe and Cu (In, GA)Se (CIGS) solar cells will be crucial. According to the studies, a markable inadequacy in V_oc_ is an important element that gives to low efficiency. The open-circuit voltage deficit (V_OC, def_) quantifies the voltage loss relative to the absorber bandgap and is defined as V_OC, def_ = E_g_/q-V_OC_, where Eg is the bandgap and is in electron Volts (eV), and V_OC_ is in Volts (V), both terms are dimensionally consistent^13^.

The CIGS devices have decreased the losses of V_OC, def_ to 350mV, whereas kesterite devices show a V_OC, def_ =589 mV^4,14^. By reducing V_OC_, both short circuit current density (J_SC_) and fill factor (FF) will also be reduced^15^, which leads to a drop in efficiency. A number of researchers have made significant efforts to improve CZTSSe efficiency^3,16^. In order to increase this efficiency, Interfacial engineering plays an important role, which can address both front and back surfaces. The front surface efforts mainly state three important areas: incorporating another buffer layer^17^, improving band bending^18^, and engaging passivation layers between the buffer and absorber^19^. Researchers explored many methods to change the front contact, such as including middle layers and making a back surface field^20^, to boost the back surface field^21,22,25^. The layer, which is included between the absorber layer & back contact, which is also known as the BSF layer, is a highly doped thin layer that is used to gain high V_OC_ in CZTSSe solar cells. When compared to conventional solar cells, the solar cells with a back surface field (BSF) layer mostly have a higher output voltage. The BSF layer increases the conduction of minority carriers to the depletion region, thereby decreasing the connection with the back surface of the cell^27^. A strong localized electric field is created in the direction of the p-n junction when a P+/P (high-low) is obtained between the BSF layer and the absorber layer. This method will decrease the dark current and the view of minority charge carriers, hence increasing the J_SC_^23^.

Many BSF materials have been utilized in order to enhance the behavior of CZTSSe-based solar cells. Rachidy et al.^24^ conducted a similarity study between SnS, CZTSSe, and CZTS as BSF to increase CZTSSe solar cell efficiency. The extreme efficiency of 17.10 has been gained by using SnS material. Shivani et al.^25^ and Kumar et al.^26^ took SnS as a BSF layer. The results pointed to the efficiency increment with the establishment of SnS and Sn2S3-based BSF layers, respectively. Mansouri et al.^27^ worked on the impact of SnSe-based BSF layer on CZTSSe solar cell, an efficiency of 20.17% is gained by an exact design. Yadav et al.^28^ used Barium Silicide (BaSi_2_) as a BSF layer in CIGS solar cells. This structure has achieved an efficiency gain of approximately 28.11% with an absorber thickness of 0.8 μm.

As a result, utilizing BaSi_2_ semiconductor as a BSF layer is an innovative method, despite the fact that several modeling studies on CZTSSe solar cells with various HTL or buffer layers have been published. In contrast to traditional architectures that only use front junction engineering, our method improves rear-side carrier selectivity and inhibits back contact recombination, two major performance bottlenecks in kesterite solar cells. In this work, BaSi_2_ is used as a BSF layer not to transport holes but to form the potential barrier that reflects minority carriers away from the back surface, reducing recombination. Barium, in its original state, is a flexible alkaline earth metal with an atomic number of 56 and a shiny grey hue. The orthorhombic BaSi_2_ is highly efficient for photovoltaic (PV) applications due to the large band gap range (1.3 eV) and excellent stability at normal conditions^29^. BaSi_2_ can be taken as an efficient back surface field material for PV technology. As a result, this study aims to assess the theoretical potential of this structure under idealized simulation conditions and provide insight into the rear interface design strategy for future experimental implementation.

The main objective of this paper is to use a less costly, sustainable, and easily accessible material to enhance the electrical and thermal performance of CZTSSe solar cells. As we know, BaSi_2_ has not been used as a BSF layer with CZTSSe solar cells, and no research publication has been published on the studies of the CZTSSe/BaSi_2_ hetero-junction. While incorporating BaSi_2_ as a BSF layer in the p-type CZTSSe solar cell helps reduce minority carrier (electron) recombination via Conduction Band Offset (CBO) it also introduces a Valence Band Offset (VBO). If the valence band of BaSi2 lies significantly lower than that of CZTSSe, a hole barrier can form at the interface. This hinders the transport of majority carriers (holes), potentially leading to hole accumulation and enhanced interface recombination.

Our important aim in this study is to find the performance of CZTSSe with the execution of a different BSF layer and provide inputs towards enhancing its electrical and thermal properties. The CZTSSe solar cell model has been examined and finalized using the *SCAPS-1D simulator*^30^, *both with and without the inclusion* of the BSF layer. The absorber layer’s thickness and doping have been adjusted, and the effect of operation temperature on the solar cell has been explored. The gathered results have been equated to empirical studies^16^. Later, BaSi_2_ was examined as a BSF layer in CZTSSe structure and correlated to other BSF materials^24^. Although V_OC_ loss is a critical challenge in CZTSSe solar cells, it primarily arises from high defect densities, interface recombination, and band tailing – rather than from temperature effects. However, elevated temperatures during operation can further degrade performance by increasing recombination rates and carrier scattering. Therefore, this work not only investigates absorber optimization but also evaluates the influence of temperature on the stability of key device parameters.

## Methodology and simulation

**Fig. 1:**
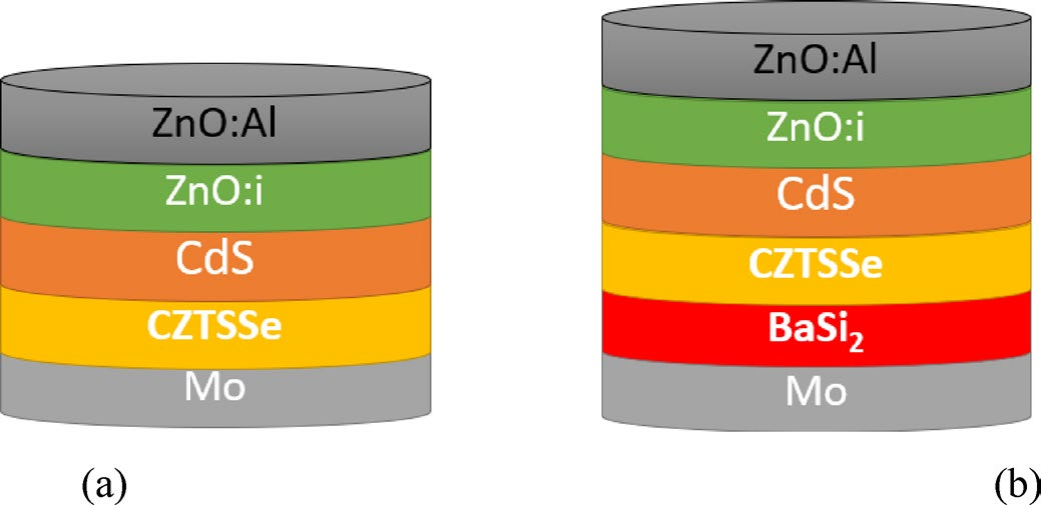
Schematic structure of solar cell (**a**) without and (**b**) with BaSi_2_ BSF layer.

Figure 1(a) illustrates the fundamental framework, which comprises all layers except the BSF layer for CZTSSe solar cells. Figure 1(b) shows the proposed structure with a BSF layer, which contains a BaSi_2_ BSF layer that is 0.3 μm in thickness and with a bandgap of 1.3eV^29^.

In recent years, mathematical calculations have stated that they have a significant advantage in kesterite-based solar cells, design, and comprehension^43^. *The Solar Cell Capacitance Simulator-One-Dimensional (SCAPS-1D) software is used in this paper*,* which is a 1D simulation tool developed by the Electronics and Information Systems department (ELIS) at Gent University*^30^. SCAPS-1D is designed to determine Poisson’s and the continuity equations for free electrons and holes, which are three fundamental equations for semiconductor physics. Table 1 shows the material statistics that were taken from both theoretical^24,28^ and experimental studies^16^, for use in the simulation.

**Table 1:** Characteristics of various layers that are contained in *SCAPS-1D*^30^.

Parameters	BaSi_2_	ZnO: i	CZTSSe	ZnO: Al	CdS
Dielectric permittivity (relative)	12.5	9	13.6	9	10
Band gap (eV)	1.3	3.3	1.097	3.3	2.4
Electron affinity (eV)	4.2	4.4	4.1	4.4	4.2
Thickness (µm)	0.3	0.1	1.8	0.2	0.05
Effective conduction band density (cm^− 3^)	10^19^	2.2 × 10^18^	2.2 × 10^18^	2.2 × 10^18^	2.2 × 10^18^
Doping concentration of donors (cm^− 3^)	0	10^18^	0	10^18^	10^17^
Doping concentration of acceptors (cm^− 3^)	5 × 10^18^	10^18^	10^15^	0	0
Effective valence band density (cm^− 3^)	4.13 × 10^19^	1.8 × 10^19^	1.8 × 10^19^	1.8 × 10^19^	1.8 × 10^19^

### Findings and analysis

The SCAPS-1D software is used to simulate the optoelectronic characteristics of the CZTSSe solar cell. This simulation states the parameters of decreasing the thickness of the CZTSSe absorber. The mathematical observations describe the effect of a BaSi2 BSF layer on the device’s performance. The work analyzed the impact of CZTSSe layer thickness, doping concentration, and temperature on photovoltaic (PV) characteristics using AM 1.5G solar spectrum. The simulation results are obtained under idealized conditions using the SCAPS-1D tool^30^, which includes low defect density, a perfect interface, and optimized band alignment. These assumptions help us to explore the theoretical potential of CZTSSe solar cells with a BaSi_2_ back surface field layer. Our objective was to investigate the best case theoretical performance of a CZTSSe device with a BaSi_2_ BSF layer under optimized conditions.

**Table 2:** Simulation and experimental results.

Cell type	CZTSSe Thickness (µm)	V_OC_ (V)	J_SC_ (mA/cm^2^)	FF (%)	Ƞ (%)
Conventional Experimental^19^	1.8	0.520	34.64	67.2	12.3
Conventional SCAPS-1D^30^ simulation	1.8	0.532		68.87	12.5

### Conventional design (Without BaSi_2_)

The CZTSSe standard design (Fig. 1(a)) was initially taken out for analysis. A correlation is being established between the outputs and the experimental CZTSSe solar cells^16^. Table 2 depicts the collected electrical parameters. The CZTSe-based solar cell efficiency is 12.54% with a JSC of 34.3 mA/cm², a VOC of 0.532 V, and a FF of 68.87%. The results coordinate well with the experimental outputs shown in^16^.

**Fig. 2:**
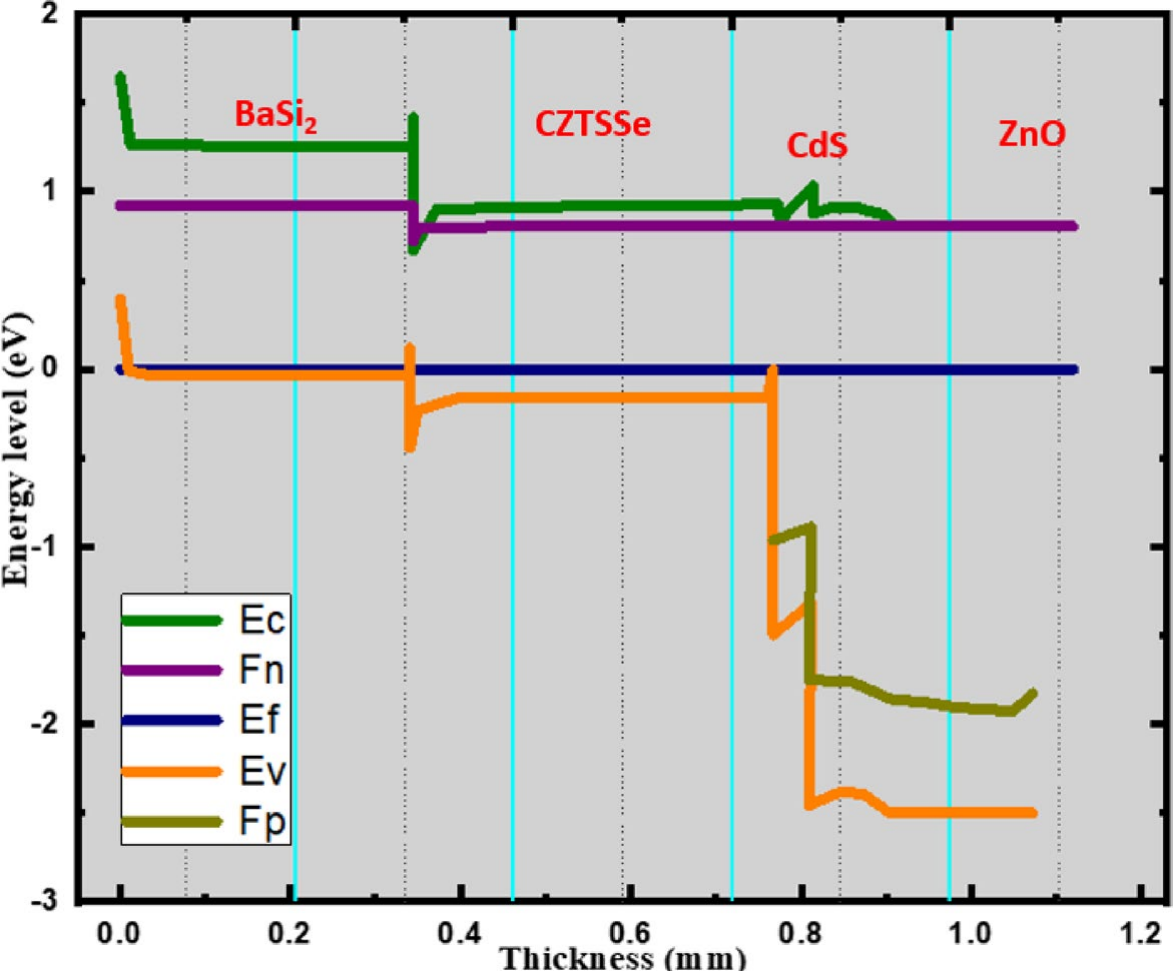
Band diagram of a CZTSSe solar cell with a BaSi_2_ BSF layer.

### New design (with BaSi_2_)

A highly doped (5 × 10^18^cm^− 3^) BaSi_2_ BSF layer is introduced at the front side of the CZTSSe absorber by creating a p-CZTSSe/p + BaSi_2_ heterojunction. The p/p + junction formed by mixing BaSi_2_ and CZTSSe holds the movement of minority carriers towards the back surface and generates a localized electric field towards the p-n junction. Therefore, there is a reduction in the amount of dark current and the reflection of minority carriers^20^. The decrease in the dark current is importantly referred to the intensified electric field maintained towards the p-n junction. Additionally, there are many other variables that contribute to this reduction: (i) Improved carrier mobility emerges from the electric field between CZTSSe and BaSi_2_, leading to decreased recombination^27^. (ii) The p-p + heterojunction of CZTSSe and BaSi_2_ generates a bigger depletion region, which increases charge separation and reduces the recombination. (iii) The BSF behaves as a photon reflector at the rear contact, improving light absorption by repelling unabsorbed photons back into the absorber layer^31^, which improves J_SC_ and helps to increase the efficiency.

The band energy diagram is shown in Fig. 2, in which the energy levels in terms of thickness are shown as a function of layer. The band alignment at the HTL/CZTSSe interface in this structure results in a cliff-like conduction band offset, which is known to promote minority carrier (electron) recombination. Ideally, the HTL should present a small spike (ΔE_C_ ≈ + 0.1 eV) to block electrons without impeding hole transport. Future studies should focus on alternative HTLs with more favorable conduction and valence band alignments to minimize interface recombination losses.

**Fig. 3:**
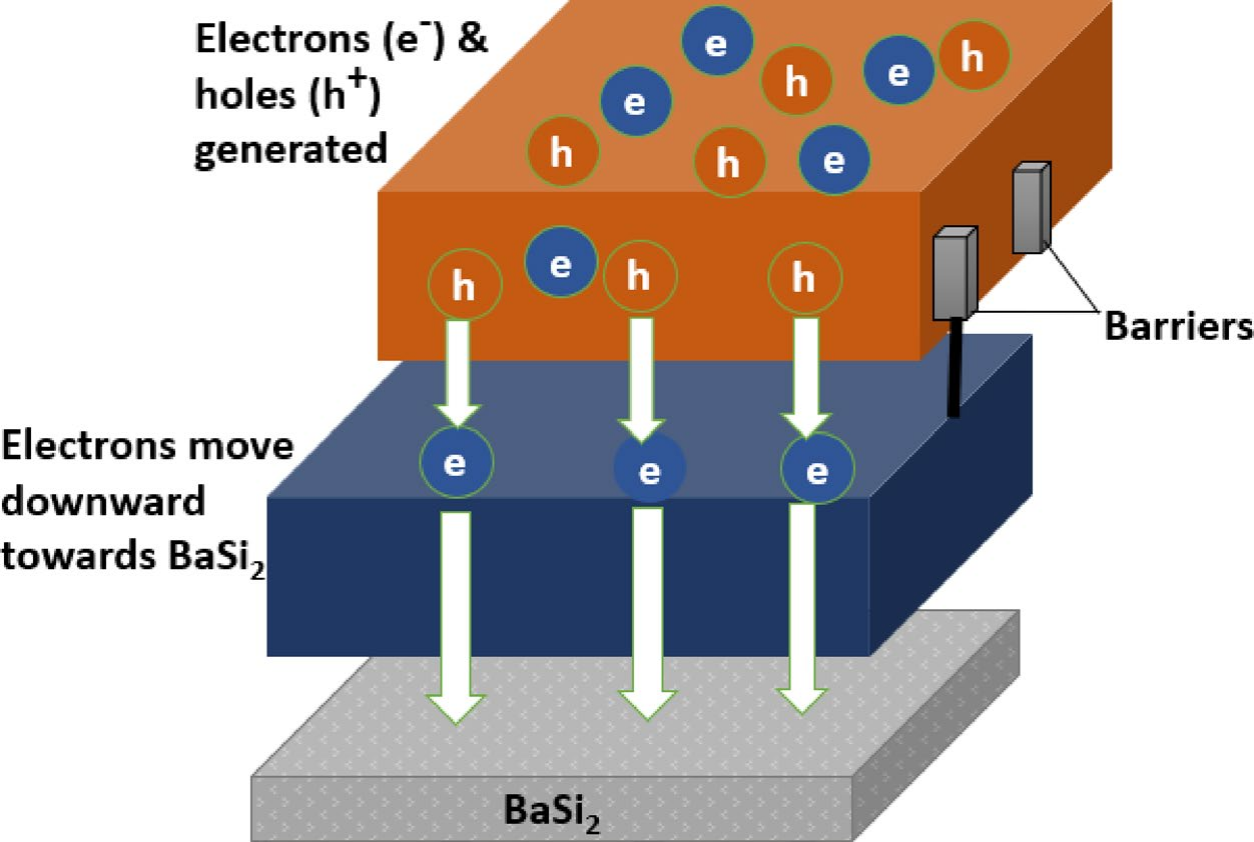
Schematic of CZTSSe solar cell with BaSi_2_ BSF layer.

Due to the rarest characteristics, the CZTSSe/BaSi_2_ heterojunction is a specifically notable finding. Firstly, the conduction band (Ec) sustains the vertical axis in a rather immediate and drastic fashion, which is like a spike, at a thickness of 0.3 μm. This Phenomenon would refer that there is a change between the conduction band levels present at the junctions of CZTSSe/BaSi_2_, which is also known as Conduction Band Offset (CBO). The state of electrons is more precisely this: they are literally opposed by some type of energy barrier in the flow from CZTSSe to BaSi_2_. Therefore, the least number of electrons can approach the back surface, thereby decreasing the number of losses due to recombination and increasing the efficiency of charge carrier collection. Along with the red line (Fn), which consists of a spike-like feature at the identical interface. The Fermi level (Fn) undergoes a sudden change, including fast injection of electrons into the CZTSSe layer. The displacement of electric charges increases the open-circuit voltage (V_OC_) and fill factor (FF). Actually, the CZTSSe/BaSi_2_ interface plays a crucial role in enhancing the efficiency of solar cells by effectively managing the displacement of charge carriers. These findings highlight the importance of utilizing well-shaped material surfaces in achieving precise power conversion efficiency. The electric field structure developed in the CZTSSe solar cell with BSF is shown in a schematic Fig. 3.

The BSF layer is taken between 0.1 and 1 μm. The recommended solar cell efficiency remains unchanged for thicknesses above 0.3 μm. Hence, it is suggested to utilize a 0.3 μm thin BaSi_2_ layer in the following parts of this study with the goal of decreasing material weight and layer thickness. Table 3 shows the photovoltaic performance parameters of the CZTSSe-based solar cell, which include VOC, JSC, FF, and efficiency. The difference is taken between the outputs gained with and without the BSF layer, and the simulation work is done by Rachidy et al.^24^, and the experimental work conducted by Hao Wei et al.^16^. When compared to the device without a BSF layer, the device using the BaSi_2_ BSF layer shows significantly improved predicted performance.

**Fig. 4:**
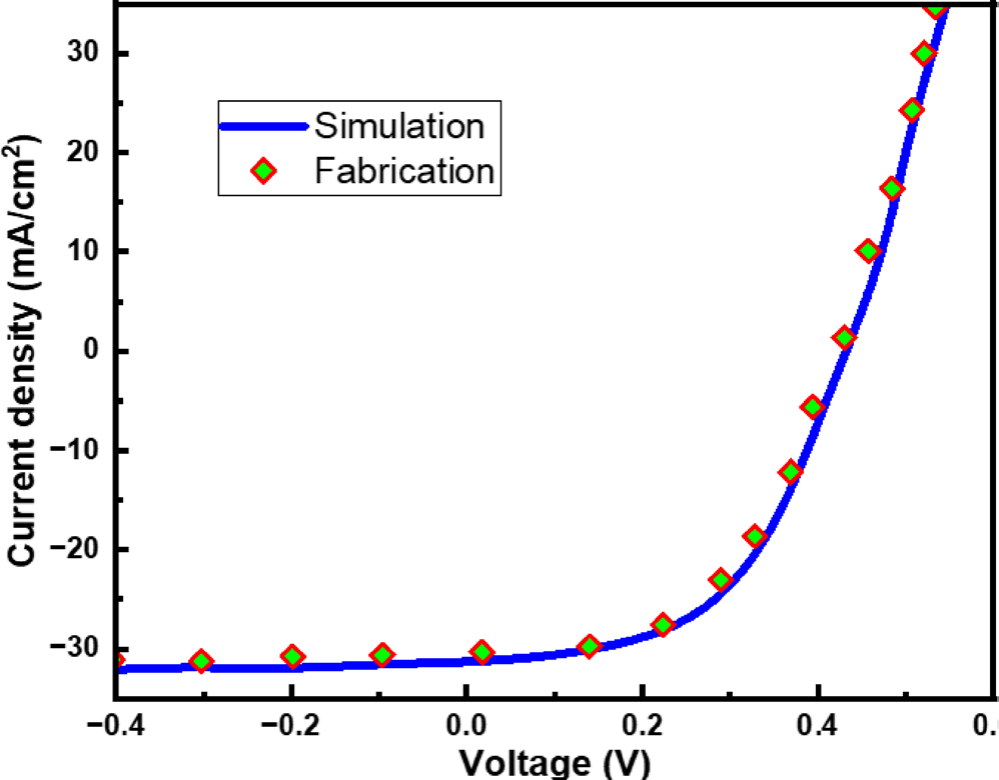
Calibration of Simulation results with experimental data^44^.

In order to calibrate our simulation results, we have simulated our device with the same dimensions presented in literature^44^, and the J-V curve is presented in Fig. 4. From this figure, it is observed that the simulation and experimental data match with each other, which validates our simulation results.

The CZTSSe solar cell showed an efficiency of 12.54%. Although the addition of a 0.3 μm BaSi2 layer showed a remarkable improvement in Power Conversion Efficiency (PCE) to 16.37% for the same 1.8 μm absorber thickness, it also showed a substantial improvement in performance. The important logic that provides this increase in efficiency is the use of both CZTSSe as an absorber layer and BaSi2 as a BSF, which leads to improved absorption of photons by the cell. The significant advantage in efficiency can be achieved through the reduced losses in recombination, promoted by the presence of the BaSi_2_ layer. The inclusion of the BSF layer resulted in a significant improvement in the JSC to 36.57 mA/cm². The 6.6% rise in J_SC_ can be attributed to the BaSi_2_ layer’s capacity to incorporate more photons, resulting in the production of more electron-hole pairs, therefore increasing the current produced by the cell. Hence, a significant number of photons can be taken, resulting in the production of more electron-hole pairs.

The V_OC_ improved from 0.532 V to 0.744 V as a result of accumulating the BSF layer. The increment of V_OC_ by 39.8% could be a result of the reduced recombination of charge carriers at the back surface, which is directly caused by the improved electric field within the cell. The FF accomplished a minimal reduction from 68.87% to 60.15%. An additional BSF layer occurred due to series resistance or other parasitic resistances, which may be the reason for the drop of FF. Although there was a reduction, the overall efficiency still improved, indicating the positive effect of the BSF layer.

### Absorber thickness effect

The work focused on a repeated numerical analysis to know the effect of changing the thickness of the CZTSSe absorber layer, varying from 0.5 to 3 μm, on the parameters of a solar cell that consists of a 0.3 μm BaSi_2_ layer as a Back Surface Field. This work highlights the significant impact of the BSF layer, enhancing the photovoltaic parameters as shown in Fig. 4. By increasing the absorber thickness to 350 to 400 nm, charge extraction can be improved even with thin absorbers, which is attributed to enhanced carrier collection, decreased recombination, and an increased electric field at the CZTSSe/BaSi2 interface, rather than absorption alone. The inclusion of a BaSi_2_ BSF layer mainly affects the important characteristics of the solar cell. As the thickness of CZTSSe decreases, a familiar pattern of increased efficiency and fill factor emerges. Usually, larger absorber layers are predictable to improve the efficiency as they have a greater capability to absorb photons. Though this scheme differs from the expected output, it is complicated. The increased efficiency obtained by taking thinner absorbers in the existence of the BSF layer, which can be attributed to the following phenomena:

(i) The BSF layer effectively reflects electrons generated by light towards the junction, therefore reducing recombination losses at the front contact. This opposing method enhances both V_OC_ and J_SC_, resulting in a complete improvement in efficiency.
(ii) The simulation outputs suggest that the BSF layer mainly reduces the recombination of minority carriers at the rear surface contact, providing an improvement in carrier gathering efficiency.

The regular CZTSSe solar cell gains an efficiency of 12.54% without a BSF layer. The efficiency of the design was significantly improved to 16.37% due to the inclusion of a 0.3 μm BaSi_2_ and 0.5 μm CZTSSe layers. The major improvement can be added to the increased absorption of photons and reduced losses due to recombination, which have occurred mainly in the BSF layer. In this work, our aim was to assess the theoretical performance potential of a CZTSSe-based device incorporating a BaSi2 BSF layer under idealized simulation conditions.

J_SC_ for the regular design is 34.3 mA/cm^2^. Including of BSF layer directed to a J_SC_ of 33.56 mA/cm^2^. Even though there was a small decrement, the complete improvement in efficiency recommends that the basic advantage of the BSF layer is in reducing recombination compared to increasing the current density. The V_OC_ for the regular solar cell is about 0.532 V. The inclusion of the BSF layer greatly improves the V_OC_ to 0.807 V, resulting in an improved design. V_OC_ increases by 51.7% due to lower charge carrier recombination at the back surface, resulting in higher cell voltage output. The fill factor attains an optimum value at an appropriate absorber thickness. When the absorber is too thin, insufficient light absorption and increased series resistance (Rs) result in a reduction in fill factor. Conversely, an excessively thick absorber leads to enhanced recombination and higher Rs, causing the fill factor to decrease again. Hence, the fill factor for the conventional design is 68.87%. The addition of the BSF layer will lead to a fill factor of 71.54%. This improvement will enhance charge carrier acquisition and reduce series resistance, dedicated to the complete improvement in efficiency.

The following factors can be used to hypothetically relate the results obtained:

The CZTSSe absorbers are experiencing voltage losses as a result of the short minority carrier lifetime^32^. Before reaching the space charge region (SCR), produced carriers are more feasible in solid absorbers^33^. Even in thinner absorbers, the BSF layer addresses this issue by reestablishing the connection between the carriers and the junction, thereby lowering recombination losses and enhancing carrier collection efficiency. There is a strong link between absorber thickness, efficiency, and carrier lifespan. When the thickness of CZTSSe is around 0.5 μm, maximum efficiency is achieved^34^. A thin absorber layer (typically 0.5 μm) can increase efficiency in CZTS and related thin-film solar cells by reducing bulk recombination, enhancing charge collection, and lowering series resistance, all of which outweigh the slight loss in optical absorption. The key is to balance optical absorption and electrical collection for the optimal thickness. This implies that using thinner absorbers in combination with a BSF layer may effectively control photon absorption and carrier accumulation without the disadvantages of longer carrier diffusion pathways in bigger absorbers.

In addition to improving performance, reducing CZTSSe thickness has significant financial and manufacturing advantages:

(i) Lowering the prices by material Optimization: Less raw material is needed when thinner absorbers are used, which lowers the total cost of manufacturing goods.
(ii) Improving Manufacturing Efficiency: Thinner films can be produced by controlling the rate at which materials are deposited. This reduces the time and energy required for the film deposition process, ultimately lowering the overall fabrication cost.
(iii) Feasibility: The production of CZTSSe solar cells is now more environmentally friendly due to the reduction in material and energy consumption, which is consistent with sustainable manufacturing requirements.

**Fig. 5:**
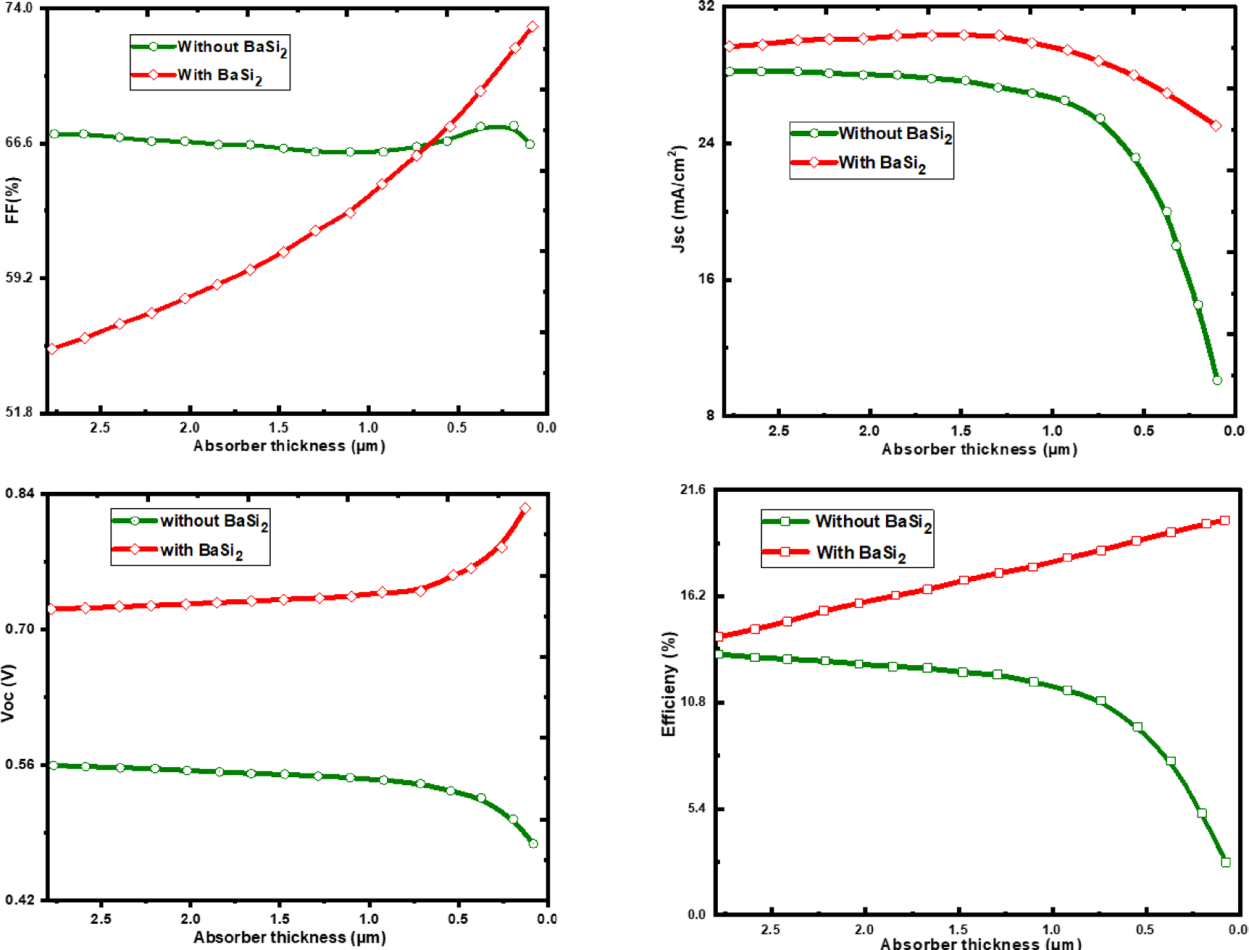
Varying the thickness of CZTSSe with without the BaSi_2_ BSF layer.

### Absorber doping concentration effect

This section presents a numerical evaluation of how various acceptor concentrations in the CZTSSe absorber layer impact the performance of the solar cell design. The solar cell design incorporated with a BaSi_2_ BSF layer with a constant density of 5 × 10^18^ cm^− 3^. Figure 5 displays the operational parameters of the solar cell. The absorber doping concentration ranges from 10^13^ to 10^18^ cm^− 3^. The analysis reveals distinct trends about the acceptor density in Voc. The Voc maintains a high value of about 0.808 V at low densities, being almost constant at acceptor densities up to 5 × 10^16^ cm^− 3^. The Voc decreases significantly at intermediate densities greater than 5 × 10^16^ cm^− 3^, reaching 0.703 V at 10^17^ cm^− 3^ acceptor density. Higher recombination rates at higher acceptor densities are likely the source of the efficiency drop, as this leads to more hole trap centers and a lower separation efficiency of photogenerated carriers.

Voc is seen to grow at acceptor densities between 10^17^ and 10^18^ cm^− 3^, reaching a value of 0.748 V. The stability of the recombination dynamics and the improvement of electronic properties at very high doping levels are responsible for the rebound observed.

There is a peak and a decline in the behavior of Jsc with respect to acceptor density. The current density (Jsc) increases significantly at low to moderate densities, peaking at an acceptor density of 10^17^ cm^− 3^. Improved carrier generation and collection efficiency, which is initially more important than recombination losses as the hole density rises, is the cause of this increase. The Jsc rapidly reduces and then stabilizes when the acceptor densities fall between 10^17^ and 10^18^ cm^− 3^. Increased Coulomb interactions cause higher rates of recombination for photo-generated electrons, which lowers the current density and causes the abrupt drop. The low minority carrier lifetimes of CZTSSe are known to restrict carrier diffusion lengths. An increase in absorber thickness beyond the diffusion length causes produced carriers to recombine before to collection, which lowers J_SC_.

Until the acceptor density reaches 10^15^ cm^− 3^, the FF stays comparatively steady, indicating that slight increases in hole density have no effect on the cell’s PCE.

After FF reaches a concentration of 10^15^ cm^− 3^, there is a noticeable decrease followed by an increase. An increase in recombination and the ensuing losses are likely the primary cause of this drop. The trend then increases at higher densities, possibly due to the electrical properties of the cell adjusting to the altered doping levels and achieving a stable performance level. However, the fill factor rises dramatically above 10^17^ cm^− 3^ because of the enhanced built-in electric field and decreased resistive losses, which increase the PCE. This impact is seen in *SCAPS-1D*^30^, when perfect interface conditions are assumed. High doping levels may cause more flaws in actual implementation, and appropriate interface passivation and defect control are necessary to achieve the true PCE benefit.

The efficiency trends are very sensitive to variations in acceptor density and closely reflect those of Jsc and FF. With an acceptor density of 10^12^ cm^− 3^, Jsc of 33.54 mA/cm^2^, Voc of 0.808 V, and FF of 72.55%, the maximum efficiency of 19.61% is reached. This optimal configuration achieves a balance between the production of carriers and the low-level incidence of recombination. As acceptor density improves, efficiency declines but stabilizes at higher concentrations (≥ 10^18^ cm^− 3^), where improved electronic properties somewhat mitigate the negative consequences of recombination.

**Fig. 6:**
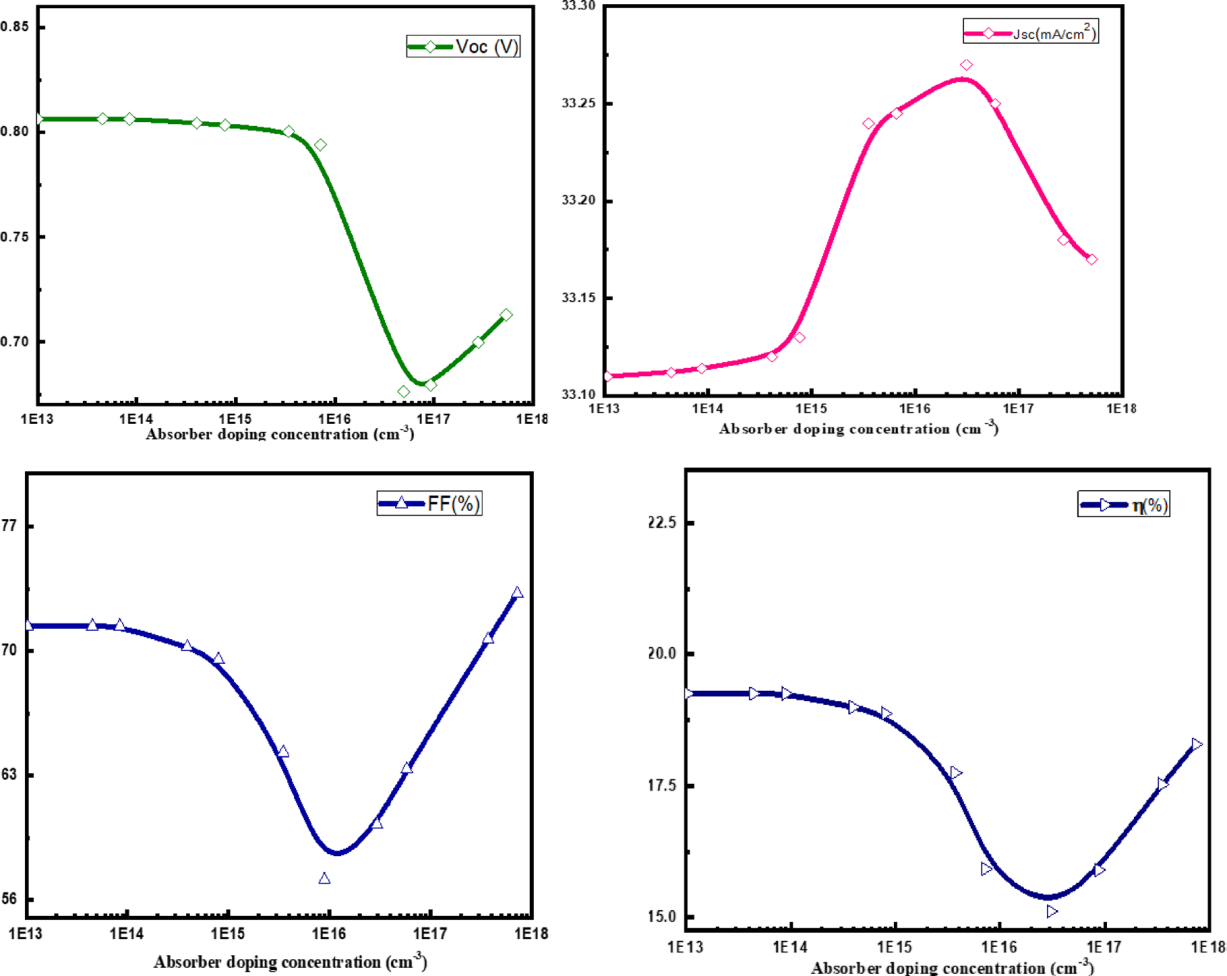
CZTSSe doping concentration effect on the proposed cell (with BSF).

### The operating temperature impact on the solar cell behavior

This part examines the efficiency of a CZTSSe solar cell that has been boosted. By keeping all the remaining specifications fixed, the temperature of the solar cell is varied between the ranges of 300 K to 400 K. As seen in Fig. 6, the investigation focuses on examining how temperature affects performance metrics. The optimized cell with a BaSi_2_ BSF layer and the regular design without one are compared in the study. In CZTS solar cells, the V_OC_ decreases approximately linearly with temperature, typically at a rate of −1.5 to −2.0mV/K. This occurs because bandgap narrowing and enhanced recombination at higher temperatures increase the saturation current, thereby lowering V_OC_. To evaluate the resilience of a device under practical working conditions, temperature analysis is beneficial.

There is a definite inverse link between V_OC_ and temperature. V_OC_ decreases as the temperature rises from 300 K to 400 K. VOC in the optimized cell initially begins at 0.808 V at 300 K and then drastically decreases as the temperature increases. The reduction in the energy band gap and the rise in reverse saturation current are responsible for this decline. Choosing a temperature range of 350 K to 400 K will make the thermal stability and performance consistency of the suggested CZTSSe/BaSi_2_ cell structure easier to understand. At a temperature of 400 K, the V_OC_ drops more quickly in the conventional design, reaching a value of 0.326 V. The enhanced temperature sensitivity highlights the benefits of the BSF layer in the revised design, which successfully maintains V_OC_ at higher temperatures. The J_SC_ shows different preferences for the two designs:

Throughout the entire temperature range, the improved solar cell’s Jsc value stays comparatively unchanged. The stability seen indicates that the BSF layer helps lessen the adverse effect of temperature on electric current generation. In the traditional design, if the temperature rises, then J_SC_ also rises from 34.3 mA/cm^2^ to 35.55 mA/cm^2^. More electron-hole pairs are produced as a result of the temperature increase, which further enhances the recombination.

As the temperature increases, FF shows a significant decline. When compared to the conventional design, the FF drops from its starting value but yet maintains significantly higher values, indicating better performance retention at higher temperatures. The optimized design performs better than the traditional design at 400 K, with a noticeably higher FF. After a slow drop, the value of FF reaches 57.64% at 400 K. The notable decline emphasizes how susceptible the conventional design is to a performance decline brought on by temperature variations.

**Fig. 7:**
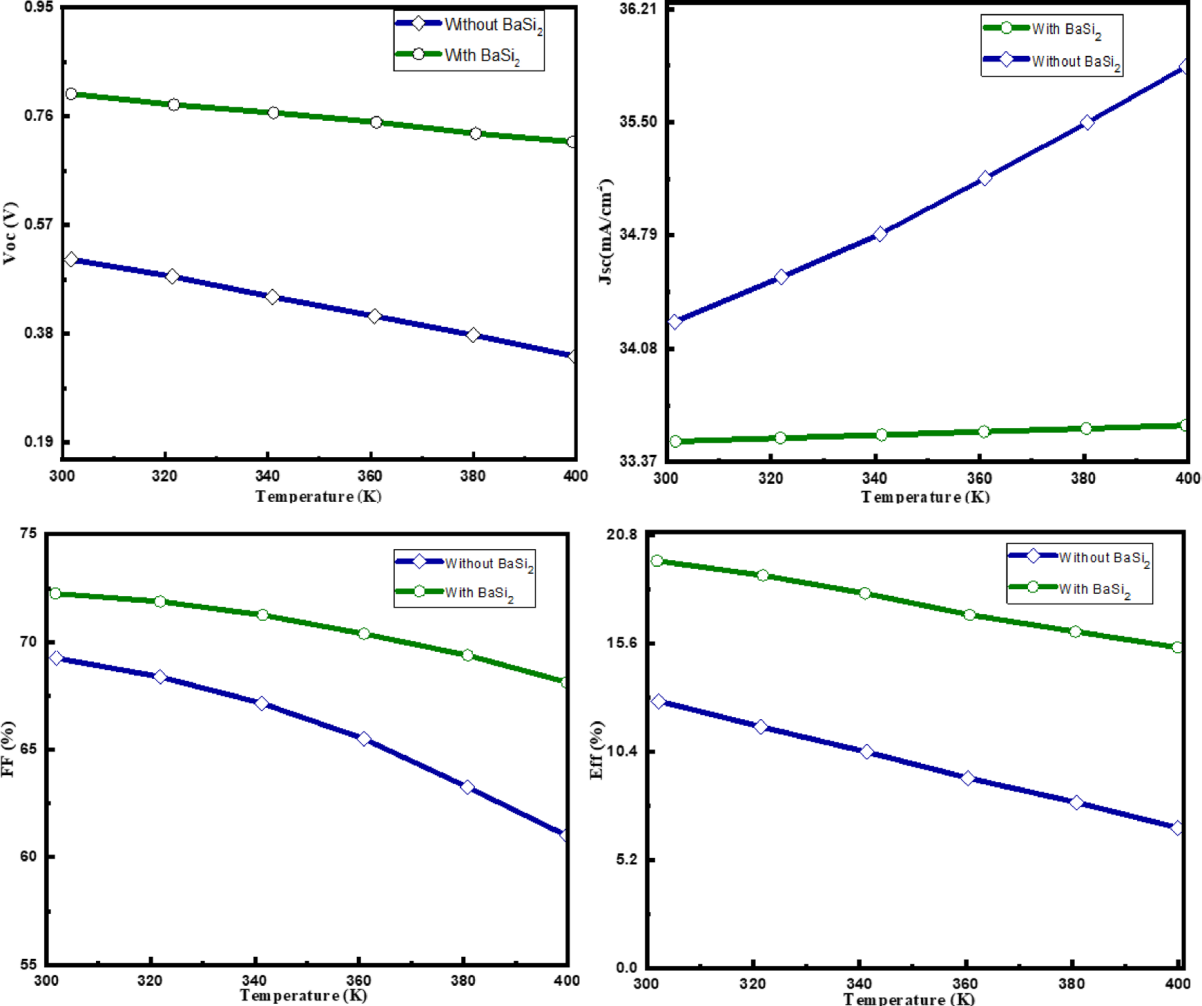
Temperature effects on CZTSSe solar cells with and without BaSi_2_.

The efficiency of the improved structure decreases from 19.61% at 300 K to 15.22% at 400 K. Despite this decrease, the improved design outperforms the conventional design at higher temperatures by a significant margin. Efficiency drops from 12.54% (300 K) to 6.67% (400 K) for the traditional design. The effectiveness of the BSF layer in the improved design, which helps to maintain exceptional performance in the face of thermal stress, is highlighted by this notable fall in efficiency. The temperature-dependent changes observed are explained by the following mechanisms:

- Voc decreases as a result of an increase in reverse saturation current brought on by the rise in intrinsic carrier concentration and the temperature-induced decrease in band gap^35^. This phenomenon is particularly noticeable in the conventional design.
- When the temperature rises, the band gap decreases, which causes the band gap to reduce. This decrease has a negative effect on Voc and FF because it speeds up the creation of electron-hole pairs while simultaneously increasing recombination^36,37^. The results show that adding a BSF layer improves efficiency at normal temperatures while also enhancing thermal stability, which is crucial in real-world situations where solar cells are subjected to temperature fluctuations. The revised design’s enhanced thermal durability may lead to improved long-term performance and reliability, making it more suitable for a range of climatic conditions. The standardized value of the efficiency V_OC_ is shown in Fig. 7. For both curves (efficiency and Voc), the CZTSSe solar cell with BaSi2 has a less steep slope than the one without.

**Fig. 8:**
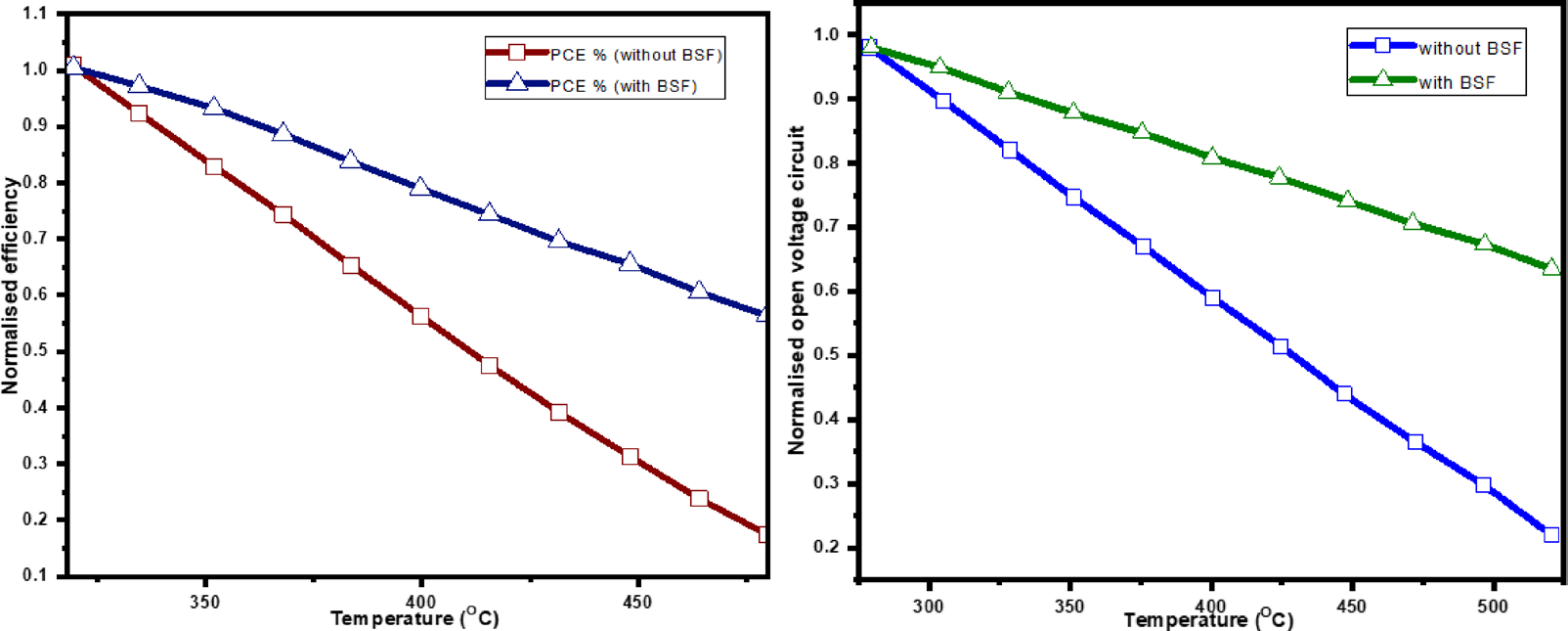
Temperature effect on the normalized efficiency and V_oc_.

### The I-V properties of the CZTSSe solar cell

Figure 8 depicts the CZTSSe-based solar cells’ J-V curves. Table 3 presents the PV properties of the basic and optimized cells, utilizing absorber layer thicknesses of 1.8 μm and 0.5 μm, respectively. These values are compared to experimental work^16^ and the standard structure simulated solar cell^24^. In comparison to the conventional design, the enhanced solar cell shows a notable increase in efficiency. The optimized cell shows approximately 18% more efficiency than the normal, bulkier cell. Performance gains can be attributed to enhanced electrical properties and reduced recombination losses in the absorber’s thinner layer.

A more financially attractive and advantageous design is suggested by the increased efficiency and the decreased amount of material used in the thinner absorber. The rise in profit is because of the decrease in expenses, combined with the 0.5 μm CZTSSe absorber material. Compared to the regular form, the enhanced cell’s Voc is 8.5% higher. Reduced recombination rates and improved charge carrier separation efficiency within the thinner absorber layer are responsible for the observed improvement. When compared to the normal cell, the optimized cell’s FF shows a notable rise of about 20.5%. The notable rise indicates enhanced charge carrier extraction and reduced resistance in the optimized design. Better device quality and higher conversion efficiency of generated power are indicated by a higher fill factor (FF).

**Fig. 9:**
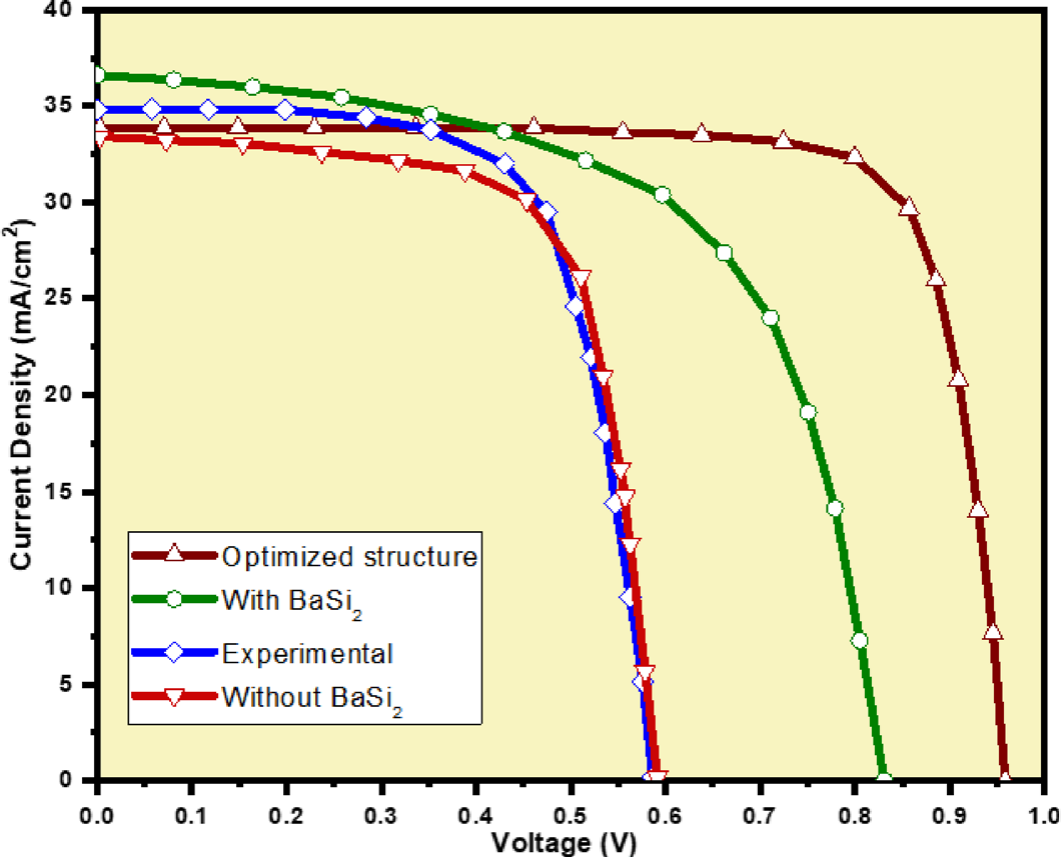
J-V appearances of CZTSSe solar cells with and without BSF.

Even though the improved cell rises in efficiency, V_OC,_ and FF, it shows a 9% reduction in J_SC_ related to the normal design. The efficiency loss is a result of the reduced thickness of the absorber layer, which controls the amount of light absorbed and, relatively, the generation of charge carriers. But there has been a reduction, the gained J_SC_ value yet stays better and more allowed, taking the complete performance improvement Fig. 9.

Table 3 presents a comparative analysis of the improved cell in relation to other theoretical and practical studies. According to both theoretical predictions and practical observations, the enhanced solar cell outperforms traditional CZTSSe solar cell designs. This design’s superior electrical properties and effective resource utilization are primarily responsible for its excellence.

**Fig. 10:**
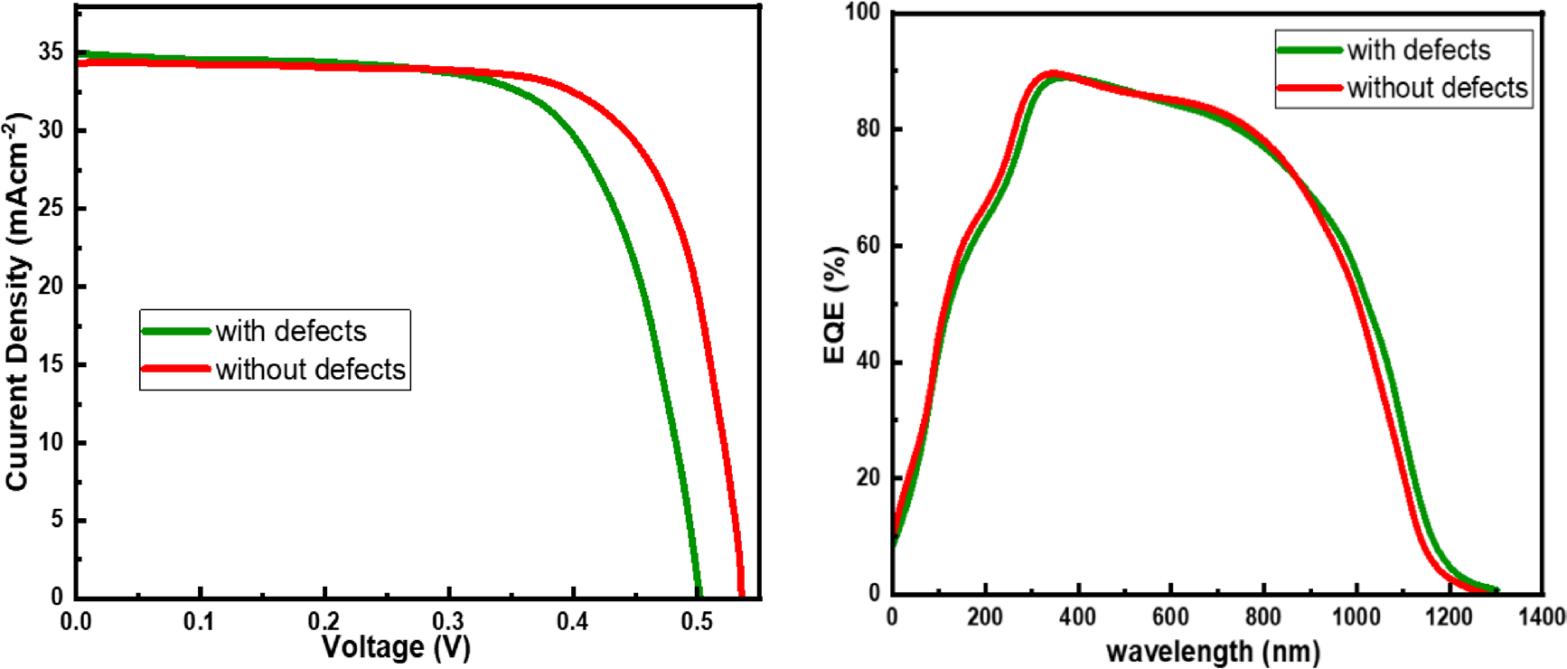
Defect parameter effect on **(a)** I-V characteristics, **(b)** Wavelength Vs Quantum efficiency.

The ability of the thin 500 nm CZTSSe absorber layer to lower production costs without losing high efficiency highlights its economic appeal and makes it a viable alternative to traditional thicker absorbers. Figure 10 illustrates the impact of defects on current density and External Quantum Efficiency (EQE). The interface defects at the CZTSSe junctions induce significant interface recombination and energy barrier mismatch, which consequently reduce the V_OC_ and FF. These defects act as recombination centers that trap photogenerated carriers, thereby limiting the separation of quasi-fermi levels and lowering the attainable V_OC_. In addition, localized states at the interface can cause the Fermi level pinning and hinder efficient carrier transport across the junction, further reducing FF and overall device performance Table 4.

### Investigating BaSi_2_ BSF potential in CZTSSe solar cells

When creating CZTSSe solar cells, a variety of materials have been employed as the back-surface field (BSF) layer, including SnS, Sn_2_S_3_^38^, SnSe^27^, CZTSe^39^, and others. Additionally, theoretical research has been done on these materials. The advantages of using BaSi2 as BSF over other materials used in CZTSSe (TFSC) are shown in Table 5. Compared to the previous BSF layers, BaSi_2_ has a higher absorption coefficient. Because BaSi_2_ is widely distributed in the Earth’s crust, it is a more affordable alternative to SnS, SnSe, or Sn2S3. Moreover, strontium (Sr) impurities can be added to achieve the desired band gap of 1.4 eV^28^.

Both the durability and the minority-carrier diffusion period are long^40^. According to the modeling results, adding a BaSi_2_ BSF layer can significantly increase the photovoltaic efficiency of CZTSSe solar cells. Additionally, the notable improvements in efficiency, Voc, and FF attained by using a BaSi_2_ BSF layer demonstrate its potential to revolutionize solar cell design. BaSi_2_ is the best material for the BSF layer of CZTSSe solar cells, according to the study’s results and the advantages mentioned in Table 5. The advantage of BaSi_2_ contributes large price benefits, combined with its eminent electrical and optical characteristics, making it the best choice for further improvement of solar cell technology. In CZTSSe solar cells, adding BaSi_2_ as a BSF layer not only increases the solar cell efficiency, but it also provides an economical and eco-friendly option.

**Table 3:** Simulation and experimental results.

Cell type	CZTSSe thickness (µm)	BaSi_2_ thickness (µm)	V_OC_ (V)	J_SC_ (mA/cm^2^)	FF (%)	Ƞ (%)
Conventional Experimental^16^ Conventional simulation Proposed (This work)	1.8 1.8 1.8	- - 0.3	0.520 0.734 0.745	34.64 39.60 37.10	67.2 80.4 60.18	12.3 12.54 16.60
Optimized (This work)	0.5	0.3	0.808	33.54	72.55	19.61

**Table 4:** Comparing the proposed cell’s PV performance to other CZTSSe-based solar cells.

*N*	Research type	CZTSSe thickness(µm)	Ƞ (%)	FF (%)	J_SC_ (mA/cm^2^)	V_OC_(V)	Ref
1	Experimental	2	12.6	69.8	35.2	0.513	^41^
2	Experimental	2.2	11.1	69.8	34.5	0.459	^40^
3	Experimental	1.8	12.3	67.2	34.98	0.521	^16^
4	Theoretical	1.8	12.54	68.87	34.30	0.532	^26^
5	Theoretical	2.5	15.6	60.48	38.61	0.65	^42^
6	Theoretical (This work)	1.8	16.60	60.18	37.10	0.745	[*]

[*] The proposed cell herein.

**Table 5:** Comparing BSF layer to previous studies.

*N*	Research type	Absorber material	BSF	PCE(%) without BSF	PCE(%) with BSF	Enhancement of PCE(%)	Ref
1	Theoretical	CZTSSe	CZTSSe	12.4	15.3	23.39	^24^
2	Theoretical	CZTSSe	CZTSSe	12.4	15.0	20.97	^24^
3	Theoretical	CZTSSe	SnS	12.4	15.7	26.61	^24^
4	Theoretical	CZTSSe	SnSe	14.36	16.44	14.48	^27^
5	Theoretical	CZTSSe	Sn_2_S_3_	12.57	17.04	35.56	^38^
6	Theoretical	CIGS	BaSi_2_	19.71	26.24	33.13	^28^
7	Theoretical (This work)	CZTSSe	BaSi_2_	12.54	19.61	56.38	[*]

[*] The proposed and optimized cell herein.

## Conclusion

In this work, the SCAPS-1D software package was used to perform numerical simulations of the CZTSSe solar cell, allowing for an analysis of its performance and potential efficiency improvements. The effects of parameters such as doping concentration, absorber thickness, and temperature on the optoelectronic performance of the cell are analyzed. A thin 0.3 μm BaSi₂ layer is introduced to modify the conventional CZTSSe device architecture, yielding an Al/ZnO/CdS/CZTSSe/BaSi₂/Mo structure. Mathematical modeling is employed to correlate the photovoltaic characteristics of the simulated cells with theoretical and experimental trends. Incorporation of the BaSi₂-based BSF layer leads to noticeable improvements in J_SC_, V_OC_, and overall efficiency, even as the CZTSSe absorber thickness is reduced. The newly improved model exhibits an efficiency of 19.61% under ideal conditions, with FF = 72.55%, V_OC_=0.808 V, and J_SC_=33.54 mA/cm². Furthermore, we intend to reproduce this work experimentally and evaluate the long-term stability of the proposed solar cell when exposed to different environmental conditions, including humidity, dust, and temperature variations.

## Data Availability

The data can be made available upon reasonable request from the authors (T.R.S.C. and D.K.P.).
